# BEST PRACTICES FOR COMMUNITY-ENGAGED RESEARCH AND PRACTICE WITH CBOS THAT ENGAGE DIVERSE OLDER ADULTS

**DOI:** 10.1093/geroni/igae098.0460

**Published:** 2024-12-31

**Authors:** Emy Haruo, Mary Mitchell, Lanvin Andres, Lesley Steinman

**Affiliations:** Neighborhood House, Seattle, Washington, United States; Aging and Disability Services - Seattle/King County Area Agency on Aging, Seattle, Washington, United States; IDIC Filipino Senior & Family Services, Seattle, Washington, United States; UW Health Promotion Research Center, Seattle, Washington, United States

## Abstract

PEARLS partner organizations will share best practices on integrating PEARLS into routine service delivery to reach historically marginalized older adults, and how they have partnered with researchers to strengthen study feasibility, equity and sustainability to translated learnings into practice. The PEARLS Equity Study guided new dissemination and implementation strategies that aligned with the strengths, priorities, and needs of community-based social service organizations (CBOs) including cultural organizations, area agencies on aging, senior centers, and family and human services agencies. New communication approaches included proactive group and 1:1 outreach to organizations; a new peer-to-peer video with PEARLS organizations sharing how they have reached underserved communities; and updated PEARLS messaging that resonated with values of CBO leadership, staff and clients. For example, new messaging included downplaying “evidence-based program” which has often been used to promote programs that were not designed with diverse older adults, and instead amplifying stories of impact that highlight both quantitative and qualitative changes in outcomes that mattered. New supports for program adoption included 1:1 support calls for tailored guidance for different CBO, staff, and community contexts; distance on-demand training with trainers from diverse CBOs to improve accessibility and sustainability; and Zoom webinars and community conversations co-led by PEARLS partners (including local partners featured during this GSA Symposium) and our research center that targeted what CBOs are looking for to address depression and disconnectedness in their communities. Our community-academic partnership and PEARLS community of practice continues to engage regularly to share adaptations as an implementation strategy for health equity.

